# Endothelial Progenitor Cells Induce a Phenotype Shift in Differentiated Endothelial Cells towards PDGF/PDGFRβ Axis-Mediated Angiogenesis

**DOI:** 10.1371/journal.pone.0014107

**Published:** 2010-11-24

**Authors:** Moritz Wyler von Ballmoos, Zijiang Yang, Jan Völzmann, Iris Baumgartner, Christoph Kalka, Stefano Di Santo

**Affiliations:** 1 Department of Cardiac Surgery, Children's Hospital Boston and Harvard Medical School, Boston, Massachusetts, United States of America; 2 Division of Vascular Medicine, Swiss Cardiovascular Center, Inselspital, Bern University Hospital, University of Bern, Bern, Switzerland; Feinberg Cardiovascular Research Institute, Northwestern University, United States of America

## Abstract

**Background:**

Endothelial Progenitor Cells (EPC) support neovascularization and regeneration of injured endothelium both by providing a proliferative cell pool capable of differentiation into mature vascular endothelial cells and by secretion of angiogenic growth factors.

**Objective:**

The aim of this study was to investigate the role of PDGF-BB and PDGFRβ in EPC-mediated angiogenesis of differentiated endothelial cells.

**Methods and Results:**

Conditioned medium from human EPC (EPC-CM) cultured in hypoxic conditions contained substantially higher levels of PDGF-BB as compared to normoxic conditions (P<0.01). EPC-CM increased proliferation (1.39-fold; P<0.001) and migration (2.13-fold; P<0.001) of isolated human umbilical vein endothelial cells (HUVEC), as well as sprouting of vascular structures from *ex vivo* cultured aortic rings (2.78-fold increase; P = 0.01). The capacity of EPC-CM to modulate the PDGFRβ expression in HUVEC was assessed by western blot and RT-PCR. All the pro-angiogenic effects of EPC-CM on HUVEC could be partially inhibited by inactivation of PDGFRβ (P<0.01). EPC-CM triggered a distinct up-regulation of PDGFRβ (2.5±0.5; P<0.05) and its phosphorylation (3.6±0.6; P<0.05) in HUVEC. This was not observed after exposure of HUVEC to recombinant human PDGF-BB alone.

**Conclusion:**

These data indicate that EPC-CM sensitize endothelial cells and induce a pro-angiogenic phenotype including the up-regulation of PDGFRβ, thereby turning the PDGF/PDGFRβ signaling-axis into a critical element of EPC-induced endothelial angiogenesis. This finding may be utilized to enhance EPC-based therapy of ischemic tissue in future.

## Introduction

Over the last couple of years, the discovery and characterization of stem and progenitor cells have opened the doors to an exciting new field in biomedicine and potentially new therapeutic options. Given the prevalence of cardiovascular disease and the tremendous interest in re-vascularisation of ischemic tissue, cardiovascular medicine has rapidly become a popular area of investigation with regard to cell-based therapy. In the meantime, many experimental studies have described the enormous regenerative and pro-angiogenic potential of endothelial progenitor cells (EPC). Additionally, clinical studies have consistently linked a low number of circulating EPCs to higher morbidity and mortality in patients with or at risk for cardiovascular diseases [1], [2]. This evidence along with the fact that EPCs circulate in the peripheral blood, from which they readily can be isolated, makes them an attractive and promising candidate for cell-based pro-angiogenic therapy of ischemic organs.

The two major mechanisms by which EPC are thought to enhance postnatal angiogenesis is by physical incorporation of the cell into a growing vascular network and by secretion of pro-angiogenic cytokines like VEGF, PDGF and other growth factors in the proximity of sprouting endothelium [3], [4], [5], [6], [7], [8]. A number of studies have shown promising results of therapeutic angiogenesis in animal models of myocardial infarction, PAOD and other ischemic diseases that were used to study the angiogenic effect of EPC transplantation [6], [7], [9]. Recently, increasing attention has been given to the capacity of EPC to support the activity and functioning of resident differentiated cells by paracrine mechanisms. Urbich et al. have shown that the combination of soluble growth factors released by EPC, such as VEGF A, stromal cell derived factor-1 (SDF-1), insulin-like growth factors-1 (IGF-1), and hepatocyte growth factor (HGF) are able to promote migration of endothelial cells (EC) and cardiac resident progenitor cells in vitro and in vivo [7].

In previous studies we have demonstrated the significance of the paracrine activity of EPC in neovascularization of various tissues and in models of different cardiovascular conditions, respectively [10], [11], [12]. Furthermore, we partially characterized the composition of EPC-derived cytokines and its systemic effects after therapeutic administration [10]. Most recently, we also demonstrated how soluble factors secreted by EPC confer strong cyto-protective properties upon differentiated endothelium through modulation of intracellular antioxidant defensive mechanisms and pro-survival signals in differentiated endothelial cells[13]. Yet, the angiogenic cytokine signaling and paracrine function of EPC are still ill-defined, and the exact underlying mechanisms leading to increase angiogenesis and sprouting of resident endothelial cells are poorly understood for the most part.

We and other investigators have shown that EPC, capable of inducing a strong angiogenic response, express and release platelet derived growth factor (PDGF-BB) in substantial amounts [4], [7], [10]. The aim of this study was to investigate the role of the PDGF/PDGFRβ axis in the interaction between EPC and differentiated endothelial cells. Specifically, we sought to determine the relation between PDGF-BB secreted by EPC and the expression of platelet derived growth factor receptor beta (PDGFRβ) in differentiated endothelium as well as the significance of the PDGFRβ for the angiogenic response. To this end, we performed a comprehensive analysis of the impact of EPC-derived cytokines on the functional and phenotypic properties of differentiated endothelial cells.

## Materials and Methods

### Ethics Statement

All protocols received full approval from the Cantonal and the Institutional Ethics Review Board at the University of Bern, Switzerland. Written informed consent was obtained from all donors.

### Cell culture and conditioned medium preparation

HUVEC were isolated from umbilical cord by collagenase digestion [14] and cultivated in complete endothelial cell growth medium (EGM-2-MV, Lonza, Switzerland) containing 5% of fetal bovine serum (FBS). All experiments were performed using cells between passages 2 to 6. The purity of endothelial cell cultures and endothelial cell characteristics were confirmed by *UEA-1* lectin binding and DiI-Ac-LDL uptake. Peripheral blood mononuclear cells (PBMC) were isolated from blood of healthy human volunteers by density gradient centrifugation with Histopaque-1077 (Sigma-Aldrich, Switzerland) as described previously [15]. Cells were plated on culture dishes coated with human fibronectin (Sigma-Aldrich, Switzerland) and maintained in endothelial cell basal medium-2 (EBM-2) (Clonetics, San Diego, CA) supplemented with EGM-2 MV SingleQuots containing 5% fetal bovine serum (FBS), human VEGF-1, human fibroblast growth factor-2 (FGF-2), human epidermal growth factor (EGF), insulin like growth factor-1 (IGF-1), and ascorbic acid. After 4 days in culture, non-adherent cells were removed and adherent cells were trypsinized and re-plated at a density of 1×106 per well through day 7 [15]. EPC were characterized by uptake of 1,1′- dioctadecyl-3,3,3′,3′-tetramethylindocarbocyanine-labeled acetylated low density lipoprotein (Harbor Bio-Products) and BS-1 lectin (Sigma) staining, as well as by flow cytometry analysis of the following surface markers: CD34, CD133, CD45, CD14, KDR, CD31, VE-cadherin (CD144) and MCAM (CD146) [6], [16], [17].

To produce human EPC-conditioned medium (EPC-CM), EPC were cultured for 72 hours under hypoxic conditions (1.5% O_2_, 5% CO_2_, 93% N_2_) in a humidified gas-sorted hypoxic incubator using growth facto-free endothelial cell basal medium-2 (EBM-2, Lonza, Switzerland) with 1% FBS. EPC-CM was then centrifuged, sterile filtered with a 0.22 µm filter (TPP, Switzerland) and stored at −80°C until use. Conditioned medium from mature endothelial cells (HUVEC-CM) was obtained in parallel and applying the same protocol, respectively. Growth factor-free EBM-2 with 1% FBS was used as control medium in all experiments. The concentration of PDGF-BB in EPC-CM was assessed by the Luminex system (Bio-Rad, Switzerland) following the manufacturer's instructions, and as published previously [10].

The effects of five different culture mediums were compared against each other in the in-vitro experiments. This included control medium and EPC-CM (as described above). Furthermore, EPC-CM supplemented with 1 µg/ml of anti-PDGFR β antibody (α-PDGFRβ, AF385, R&D Systems, UK), and control medium containing recombinant human PDGF-BB (rhPDGF-BB) in a final concentration of 100 pg/ml and 100 ng/ml, respectively, were compared. In order to establish specificity of the observed effects for PDGFRβ-mediated signaling we additionally used an inhibitor of the PDGF-receptor kinase (AG1296, Merck) for the proliferation and migration assay at a concentration of 1 µM following manufacturer's instruction.

### Survival/proliferation assay

HUVEC were seeded into 96-well plates coated with 1% gelatin at a density of 5×10^3^/well and cultured in control medium for 24 hours before experiments. Thereon, culture medium was replaced by the experimental culture mediums and cells were cultured for further 24 hours. Finally, the number of viable cells was assessed by use of the CyQuant® NF kit (Molecular Probes, Switzerland). The proliferation rate was expressed as relative values standardize to the control group.

### Migration assay

HUVEC migration was analyzed using Costar® transwell inserts with 8 µm polycarbonate filters (Corning, The Netherlands) in 24-well plates coated overnight and at 4°C with a 1% gelatin solution containing 1 mg/ml fibronectin. One hundred and fifty µL of different medium was placed in the lower chamber of the trans-well system and 5×10^4^ HUVEC in control medium were placed above the filter and incubated for 12 hours at 37°C. Thereafter non-migrated cells were removed from the system. The migrated cells were fixed in 4% PFA for 20 min at 4°C and stained with crystal violet. The cell number was counted in 4 random high power fields and expressed as values standardized to the control group.

### In vitro cord structure formation

*In vitro* formation of EC cord structures was assessed using cell culture on growth factor reduced Matrigel™ (Becton Dickinson, Switzerland). Forty-thousand cells per well were resuspended in EPC-CM and the other experimental culture mediums, respectively, and seeded on the polymerized Matrigel™ layer. Endothelial cell cord structure formation was assessed at 8 hours of incubation. Digital microphotographs were taken from three randomly selected high power fields and cord structure formation was assessed measuring the total length and number of sprouts with the aid of ImageJ (http://rsb.info.nih.gov/ij/).

### Ex vivo aortic ring assay

Aortas from 2-month-old Wistar rats were isolated, flushed with PBS solution to remove blood, and freed from adventitial tissue. Aortic rings of 1-mm thickness were placed individually in 24-well plates coated with growth factor reduced Matrigel™, and incubated at 37°C for 5 days using the five different culture mediums [18]. Quadruplicates were performed for each culture condition and quantified as suggested by Blacher et al.[19] In brief, pictures of the aortic rings and the sprouting cells surrounding it were taken after 5 days in culture at 10× magnification and from each of the four quadrants of the aortic ring, respectively. The two maximal extensions of the tubular structures sprouting from the aortic ring wall were measured in each quadrant. Average sprout length was calculated as the mean of the four samples per condition and eight values obtained from each ring.

### Assessment of PDGFRβ expression on HUVEC

The expression of PDGFRβ on HUVEC after exposure to EPC-CM, HUVEC-CM or control medium culture was measured by means of western blotting and real-time PCR. Both total (AF385, R&D Systems, UK) and phosphorylated PDGFRβ (07-021, Millipore, Switzerland) levels were measured by use of respective antibodies. The results were normalized to the expression level of α-tubulin (Sigma-Aldrich, Switzerland) and expressed as relative values in comparison to the control group. Total mRNA isolated from HUVEC (RNeasy® Mini Kit, Qiagen, Switzerland) was transcribed to cDNA (SuperScript® VILO™ cDNA Synthesis Kit, Invitrogen, Switzerland). The amount of PDGFRβ mRNA was quantified by real time PCR using Taq primer (Hs00387364, Applied Biosystems, Switzerland). The results were normalized to the mRNA level of housekeeping gene glyceraldehyde 3-phosphate dehydrogenase (GAPDH, Hs99999905, Applied Biosystems, Switzerland) and expressed as relative values compared to the control group.

Dual-color Flow cytometric analysis was used to verify the absence of smooth muscle cell contamination in our HUVEC cultures and in order to verify the cell surface expression of PDGFRβ after EPC-CM stimulation for 24 hours. To this end, cells were fixed in 4% PFA for 10 min at 37°C, permeabilized in 90% methanol for 30 min on ice, and stained with both α-PDGFRβ (AF385, R&D Systems, UK) and anti Von Willebrand factor (vWF) antibody (AB7356, Millipore, Switzerland). The percentage of vWF/PDGFRβ double positive fraction of HUVEC was measured individually in cells cultured in control medium, EPC-CM, and control medium containing 100 pg/ml or 100 ng/ml rhPDGF-BB.

### Statistical analysis

All experiments were performed in at least triplicates. If not otherwise stated, data are presented as mean ± standard error of the mean (SEM). Unpaired Student's t-test and one-way ANOVA with Scheffe's test for posthoc comparison were used to compare group means, after testing for normality and equal variance of the data. RT-PCR data were analyzed using REST^©^ - Relative Expression Software Tool (http://www.wzw.tum.de/gene-quantification/) to compute relative differences in expression levels, and the Bonferroni correction was applied for multiple testing. All other statistical analyses were carried out in STATA (Stata Corporation, College Station, TX, Version 10.1 for Apple). Statistical significance was inferred at a 2-sided P≤0.05.

## Results

### Hypoxia increases the secretion of PDGF-BB from EPC

Previous studies have shown that the expression of paracrine factors released by EPC including VEGF and FGF is significantly induced by hypoxia [20], [21]. We determined the release of PDGF-BB by EPC subject to hypoxia in comparison to cells cultured under normoxic conditions. EPC-CM obtained from cultures in hypoxia (111.6±27.0 pg/ml) showed a five-fold increased level of PDGF-BB as compared to normoxic cultures (19.9±2.2 pg/ml, P*<*0.01).

### The PDGFRβ signaling axis is critical for EPC stimulated angiogenic activity of HUVEC

**Figure 1:**
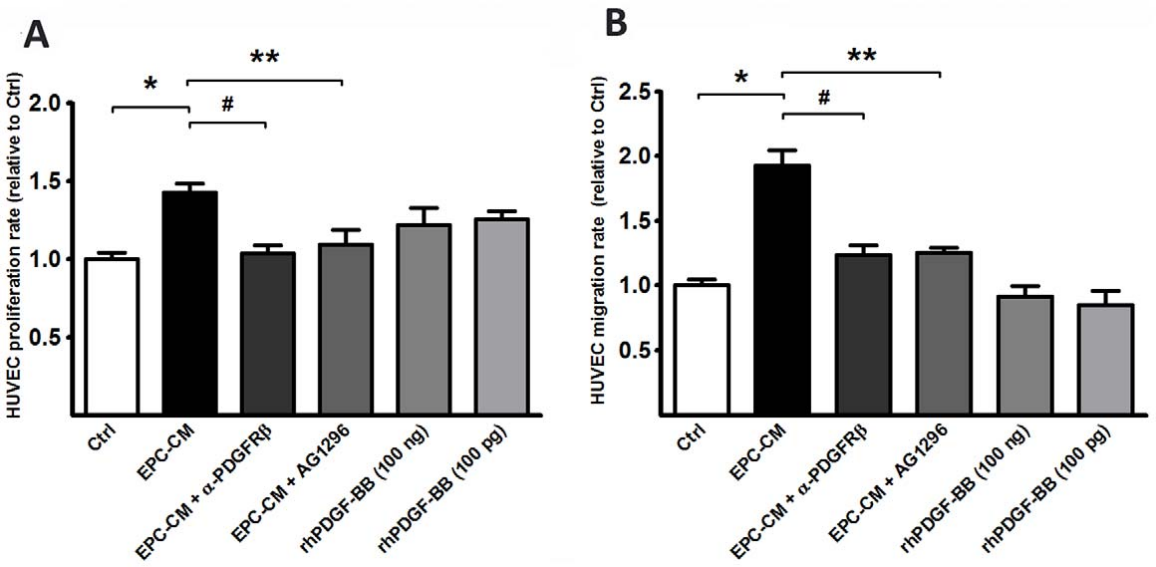
EPC-CM promotes HUVEC proliferation and migration. EPC-CM incubation significantly increased proliferation (A) and migration (B) of HUVEC in comparison to control medium. Blocking PDGFRβ with either a PDGFRβ neutralizing antibody or PDGF receptor kinase inhibitor (AG1296) offset the chemotactic and proliferative response of HUVEC to EPC-CM in both proliferation and migration. However, addition of recombinant human PDGF-BB at a similar content of EPC-CM (100 pg/ml) or 1000-times higher (100 ng/ml) was not able to promote significant proliferation or migration of HUVEC. *, P<0.0001; #, P<0.0001; **, P<0.05.

In vitro EPC-CM increased HUVEC proliferation 1.43±0.06 -fold (P<0.001) and EC migration along the cytokine gradient by a factor of 1.93±0.12 (P<0.001) as compared to controls (Figure 1A–1B). When EPC-CM was used but supplemented with the PDGFRβ neutralizing antibody AF385 migration of EC along the EPC-CM gradient was no longer different from controls (1.23±0.08, P = n.s.). Furthermore, HUVEC proliferation with EPC-CM in presence of the neutralizing antibody AF385 was decreased significantly (1.04±0.05, P = n.s.). Likewise, the treatment with PDGF-receptor kinase inhibitor AG1296 (1 µM) effectively damped the migration of HUVEC (1.25±0.04, P = n.s.) and attenuated the proliferation of HUVEC under EPC-CM culture (1.09±0.09, P = n.s.) (Figure 1A–1B).

In order to test whether PDGF alone is sufficient to elicit this angiogenic activity in differentiated endothelial cells, HUVEC were exposed to control medium supplemented with rhPDGF-BB at a similar concentration as found in EPC-CM (100 pg/ml) or at a 1000-fold higher concentration (100 ng/ml). None of these two concentrations of rhPDGF-BB alone were able to induce an increase in proliferation or migration of HUVEC (P>0.05, Figure 1A-B).

**Figure 2:**
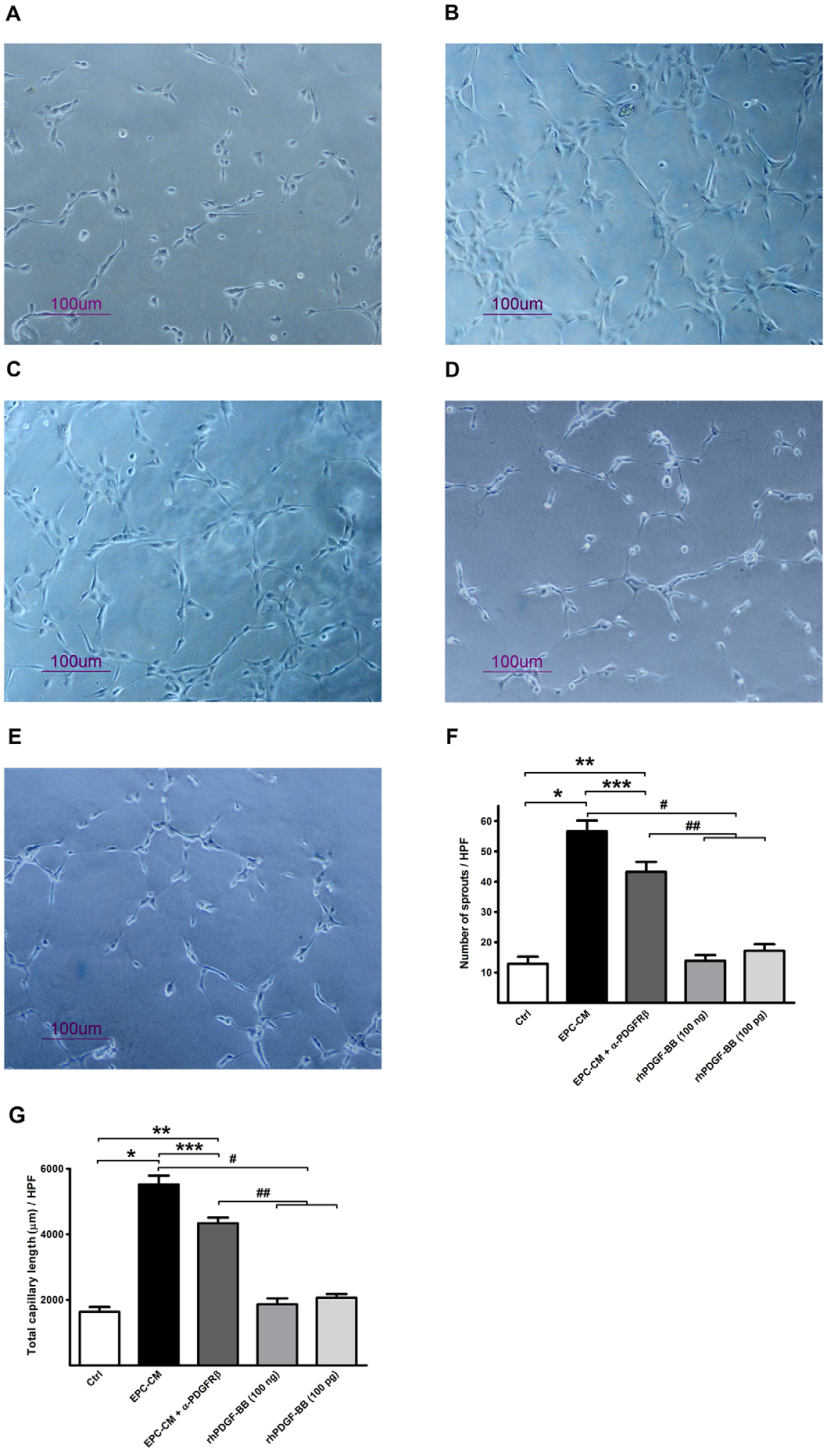
Angiogenic potential of EPC-CM on HUVEC matrigel cord structure formation. EPC-CM incubation for 8 hours strikingly accelerated the formation of EC cord structures on growth factor reduced matrigel™ (**B**) as compared to control medium incubation (**A**). Antibody mediated PDGFRβ neutralization partially inhibited the EPC-CM accelerated cord structure formation (**C**). rhPDGF-BB conditioning of control medium in either 100 ng/ml (**D**) or 100 pg/ml (**E**) concentration did not have significant effect on promoting the formation of EC cord structures. The difference effect between groups was evidenced by both the number of spouts (**F**) and the total cord structure length (**G**). * and **, P<0.0001; # and ##, P<0.0001; ***, P<0.05.

Consistently with the above findings, exposure of HUVEC to EPC-CM resulted in a significantly increased number and length of EC cord structures in growth factor reduced Matrigel™ as compared to control medium (56.63±3.56 vs. 12.88±2.36/HPF in sprout numbers, 5521±268.3 vs. 1636±1448.7 µm/HPF, P<0.0001). Again, blocking of PDGFRβ by AF385 caused partial inhibition of the EPC-CM effect (43.25±3.30/HPF in sprout numbers, 4338±170.2 µm/HPF; P<0.001 compared to EPC-CM group) whereas rhPDGF-BB supplementation of control medium did not increase the formation of endothelial cord structures in either the 100 pg/ml or the 100 ng/ml concentration (Figure 2).

**Figure 3:**
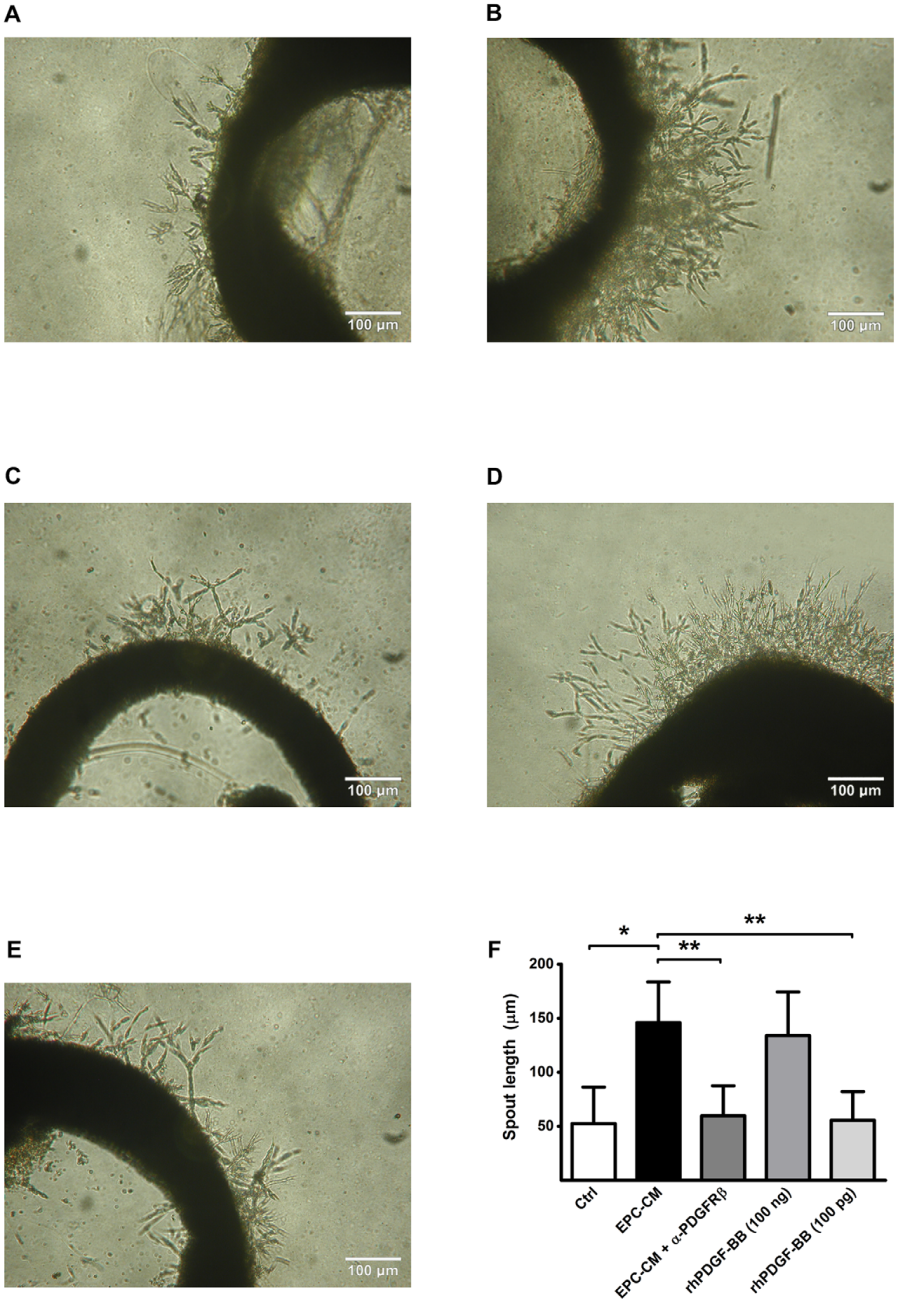
Angiogenic potential of EPC-CM on *ex vivo* aortic ring assays. Incubation with EPC-CM (**B**) enhanced the formation of vascular outgrowth from 1 mm rat aortic ring embedded in growth factor reduced-Matrigel™ compared to control medium incubation (**A**). This enhanced EC cord structure outgrowth could be blocked by the addition of 1 µg/ml PDGFRβ antibody into EPC-CM (**C**). A similar vascular sprouting extent could only be observed by stimulation aortic ring with 100 ng/ml rhPDGF-BB (1000-times concentrated than the content in EPC-CM) (**D**), but not with the concentration at 100 pg/ml (**E**). The extents of vascular outgrowth were quantitatively analyzed and presented by the length of the sprouts (**D**). *, #, P<0.001.

Furthermore, in the aortic ring assay, extensive sprouting occurred when the aortic rings were cultured in EPC-CM (145.93±7.69 vs. 52.48±9.76 µm in control, P<0.001; Figure 3B) and this sprouting was lowered in the presence of the PDGFRβ neutralizing antibody AF385 (59.78±7.99 µm, Figure 3C). A high-dose supplementation of control medium with rhPDGF-BB (100 ng/ml) showed results comparable to EPC-CM in this assay (133.95±11.66 µm, P<0.001; Figure 3D-E).

### EPC-CM up-regulates PDGFRβ expression on HUVEC

**Figure 4:**
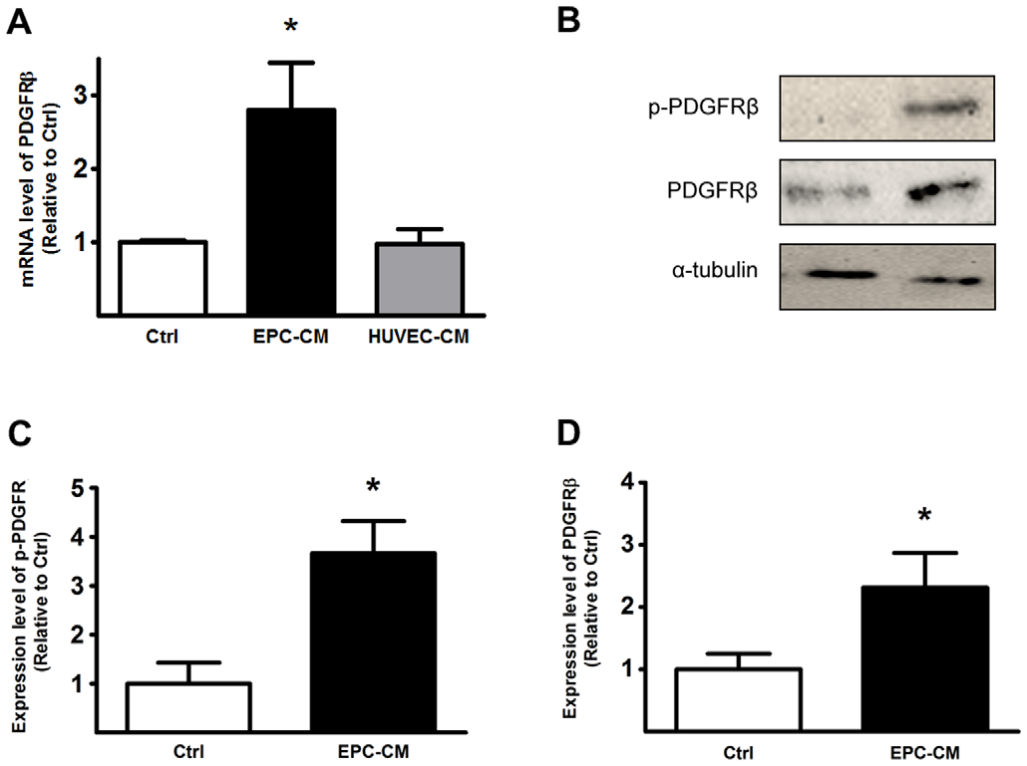
EPC-CM induces PDGFRβ expression in HUVEC. mRNA expression of PDGFRβ was determined by real-time PCR of total RNA obtained from HUVEC cultured in EPC-CM or control medium. EPC-CM incubation promoted a 2.8-fold over-expression of PDGFRβ mRNA in HUVEC compared to control medium incubation. Incubation with HUVEC-CM did not change mRNA expression levels of PDGFRβ as compared to control medium. (**A**). Protein level of PDGFRβ was analyzed by Western blot (**B**). The expressions of total PDGFRβ (**C**) as well as its phosphorylated form (**D**) were significantly up-regulated upon incubation with EPC-CM. *, P<0.05.

**Figure 5:**
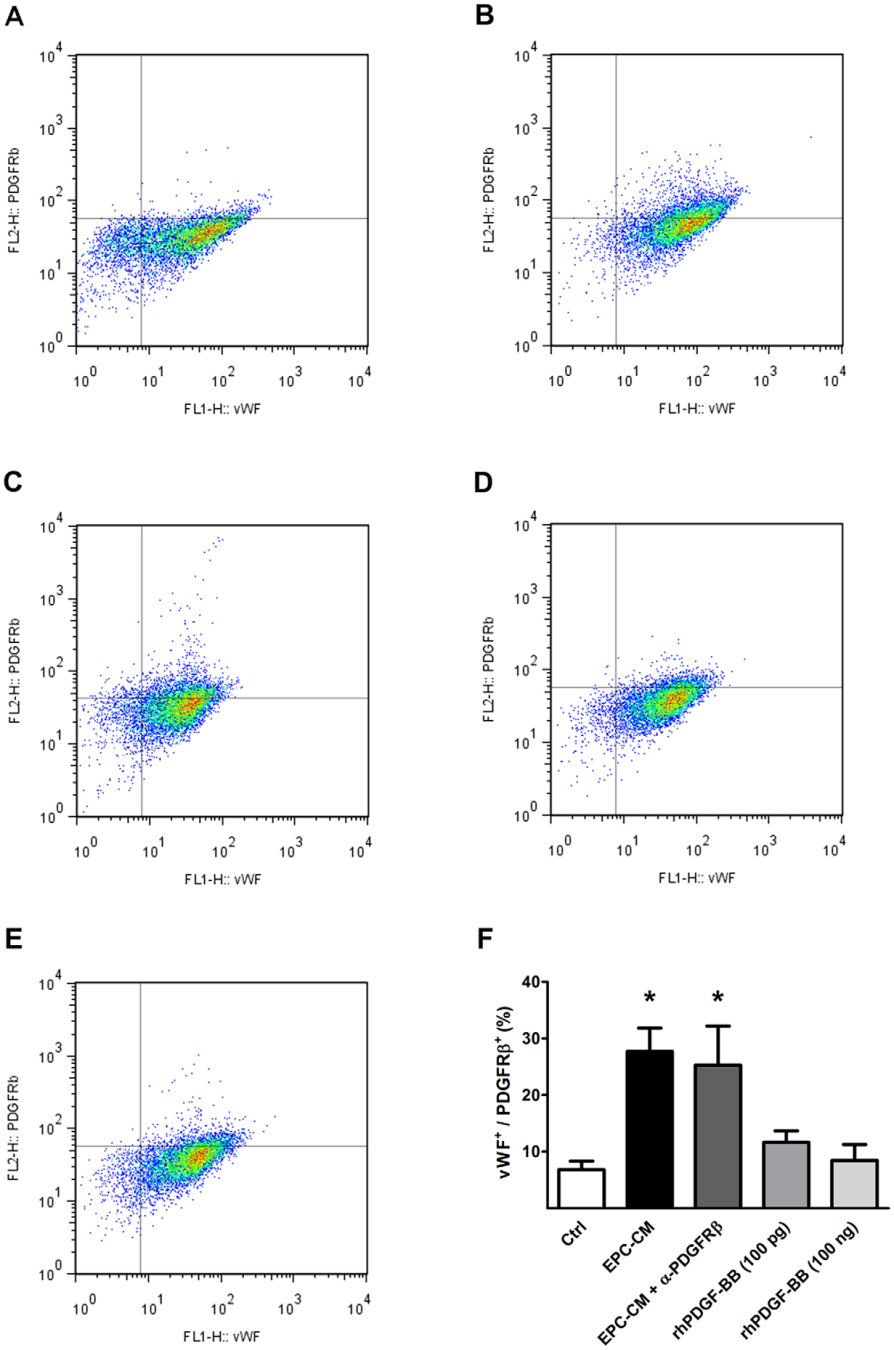
Co-expression of EC marker and PDGFRβ after EPC-CM incubation. Co-expression of vWF and PDGFRβ was measured using dual color FACS analysis. HUVEC were kept in control medium containing only 1% FCS or EPC-CM for 24 h before the measurement. HUVEC incubated in control medium showed only a fractional amount of vWF^+^/PDGFRβ^+^ cells (**A**). After EPC-CM exposure the proportion of vWF^+^/PDGFRβ^+^ double positive population was significantly increased, suggesting a strong phenotype shift of endothelial cells towards PDGFRβ^+^ (**B**). The addition of neutralizing antibody AF385 did not block the upreguation of the PDGFRβ by EPC-CM stimulation (**C**). However, such enhanced PDGFRβ expression could not be evoked by solely adding 100 ng/ml (**D**) or 100 pg/ml (**E**) rhPDGF-BB to the control medium. *, P<0.01 compared to controls.

To further investigate the signaling pathway underlying the above findings suggesting that PDGFRβ plays a major role in the EPC-CM stimulated angiogenic response of differentiated EC, we measured the transcriptional and translational levels of PDGFRβ in HUVEC. Upon EPC-CM stimulation, the mRNA level of PDGFRβ was up-regulated 2.8±0.6 -fold compared to control medium (P<0.05, Figure 4A). At the same time, conditioned medium obtained from differentiated endothelial cells (HUVEC-CM) did not change the expression levels of PDGFRβ. Western blotting also showed a significantly increased amount of both total PDGFRβ (2.31±0.56 -fold, P<0.05) and its phosphorylated form (3.67±0.66 -fold, P<0.05) expressed by HUVEC when incubated with EPC-CM (Figure 4B-D). Moreover, FACS analyses revealed a clear shift of HUVEC from a vWF^+^/PDGFRβ^−^ towards a vWF^+^/PDGFRβ^+^ double positive phenotype after EPC-CM incubation (Figure 5A-B, E; P<0.001). Neutralizing the PDGFRβ bioactivity with AF385 had only marginal effect on blocking the stimulation by EPC-CM, and resulted in a similar upregulation of the PDGFRβ (Figure 5C, E; P<0.01). However, such an increased expression of PDGFRβ in HUVEC could not be elicited by adding 100 pg/ml or 100 ng/ml of rhPDGF-BB to the control medium (Figure 5C-D, E).

## Discussion

In this study we sought to determine what role PDGF-BB and PDGFRβ play in angiogenesis as part of the interaction between EPC and differentiated endothelial cells. In an analysis of secreted cytokines we found that PDGF-BB is released by EPC in substantial quantities. Moreover, we found that incubation of EPC in reduced oxygen tension leads to a significant, more than fivefold increase in release of PDGF-BB as compared to normoxic conditions. These findings are in agreement with the observations of enhanced release of angiogenic factors by different cell types including hematopoietic stem cells and EPC [10], [20], [21]. Given the reasonably high levels of PDGF-BB we were interested to elaborate the impact of this particular cytokine on differentiated endothelial cells and its potential implications for angiogenesis.

In multiple tissue culture assays of angiogenesis the exposure of endothelial cells to EPC-CM resulted in increased proliferation, migration and organization of the cells into cord structures. All of these effects were effectively inhibited in presence of either a neutralizing anti-PDGFRβ antibody or a specific inhibitor of the PDGFRβ tyrosine kinase (AG1296) in endothelial cells. The latter is a tyrosine kinase blocker which selectively inhibits the platelet-derived growth factor (PDGF)-receptor kinase and the PDGF-dependent signaling including PDGF dependent transactivation of P2Y and MAPK [22]. Notably, supplementation of control medium with even high concentrations of PDGF-BB achieved only minimal angiogenic effects in all experimental settings that we tested. In conjunction with the increased expression of PDGFRβ that we found in EC after incubation with EPC-CM these findings strongly suggest that endothelial cells react in a hitherto undescribed fashion to cytokines released by EPC. Notably, conditioned medium obtained from differentiated EC, and rich in PDGF-BB, was not able to induce expression of PDGFRβ in EC. Hence, our data strongly indicate that EPC induce a phenotype change of EC towards increased expression of PDGFRβ and sensitivity to PDGF as a pro-angiogenic growth factor. This finding is in line with our previous report that demonstrated a distinct capability of EPC to induce and modulate strong cyto-protective properties in differentiated endothelium through modulation of intracellular antioxidant defensive mechanisms and pro-survival signals [13].

Angiogenesis is a complex process involving multiple cell lines and cytokines that encompasses two major steps, sprouting and proliferation of the endothelium resulting in formation of a primitive vascular plexus and maturation of the latter which includes the incorporation of perivascular cells into the forming vasculature [3]. The role of EPC in postnatal angiogenesis is currently under intense investigation. Multiple lines of evidence suggest that EPC contribute to angiogenesis and vascular repair by proliferation and differentiation into mature EC (structural component) as well as by secretion of a variety of cytokines (paracrine component) impacting on the fate and function of different cell types including endothelial cells, perivascular cells and progenitor cells [4], [5], [6], [7], [9]. In spite of recent advances in the field the exact composition of factors released by EPC and their relevance in angiogenesis are still poorly understood. However, several investigators have demonstrated the complexity and potency of the EPC secretome that includes many angiogenic factors such as VEGF, SDF-1 or IGF-1 [7], [23]. Our data are in line with these findings and add further evidence that EPC convey critical, angiogenic signals upon differentiated endothelial cells.

PDGF is a well-characterized trophic factor crucial for the survival and functioning of various cells including pericytes and SMC. It is also known that PDGF-BB is expressed by the endothelium nourishing the adjacent perivascular cells [3]. Furthermore, the expression of PDGF receptors has been reported in hemangioblast precursors where it accelerates the differentiation of endothelial cells [24]. However, PDGFRβ has not typically been associated with endothelial cells susceptibility and capability to participate in postnatal angiogenesis. Hence, it is an obvious anticipation that PDGF-BB secreted by EPC increases recruitment and proliferation of perivascular cells in tissues undergoing active angiogenesis. The finding that PDGF-BB is expressed in the EPC secretome, increases endothelial cell migration and proliferation as well as cord structure formation is intriguing, and given the distinct inhibition of those processes by blocking of the PDGF/PDGFRβ signaling axis strongly suggests a critical role of this signaling axis for endothelial cells in the immediate EPC:EC interaction. More specifically, our data indicate that the PDGF/PDGFRβ axis is a critical modulator of the angiogenic response evoked in endothelial cells by EPC. Inhibition of PDGFRβ drastically mitigated the angiogenic response of EC exposed to the EPC secretome but did not abolish it fully. At the same time, PDGF-BB alone was not sufficient to promote an angiogenic behavior in endothelial cells. Together our results provide evidence that EPC-CM activates multiple pathways while PDGF alone is not sufficient to trigger an effective angiogenic response in EC. These findings could probably best be explained by modulation of classic angiogenesis signaling pathways (e.g. VEGF/VEGFR-2) by the PDGF/PDGFRβ axis. However, this specific, novel hypothesis warrants further investigation.

Although, endothelial cells usually do not express PDGFRβ in high quantities and the relevance of this receptor is unclear in resting endothelium, the current literature is provides evidence that PDGFRβ is up-regulated under circumstances of increased angiogenesis [25], [26]. In order to explore this potential explanation for our observations, we determined expression of PDGFRβ in the endothelial cells exposed to EPC-CM. Indeed, we found significantly increased gene and protein expression of PDGFRβ as well as an increased phosphorylation of the receptor in endothelial cells that were exposed to EPC-CM. Also, we show that differentiated cells continue to express EC marker while the expression of PDGFRβ on EC increases. Our finding that conditioned medium from differentiated EC is not capable of triggering the same response as EPC-CM underlines the unique composition of the latter. It also suggests that the phenotype change towards PDGF-sensitivity of EC could not occur through an autocrine mechanism alone, but rather requires the stimulus from EPC. In other words, we demonstrate that EPC-CM is capable of inducing a change in endothelial phenotype towards increase sensitivity for PDGF, rending this receptor potentially an effective modulator of endothelial function in the process of angiogenesis. This theory would be consistent with several clinical trials that have shown a significantly increase in angiogenic response when cytokines like VEGF are combined with PDGF [27], [28], although the impact on perivascular cells certainly is a major reason for the benefits of a combined therapy.

At this point we do not have a definitive answer as to what exactly in the cytokine cocktail from EPC causes this phenotype change in endothelial cells, and further studies will be needed to address this specific question in detail. Also, a limitation of this study is the focus on a single cytokine and its receptor. This must be taken into consideration when interpreting the presented data. We cannot preclude, that other factors released by EPC have similar effects on the endothelium as shown here for PDGF-BB/PDGFRβ. Given the complexity of the EPC secretome and the interaction between EPC paracrine factors and the endothelium, it can be assumed that the PDGF-BB/PDGFRβ axis is just one of several pathways involved. Thus, we advocate the need for future studies to clarify the EPC:endothelium interactions in more detail.

In summary, we sought to elucidate the role of PDGF and PDGFRβ in the interaction between EPC and resident EC and we found evidence for a change in endothelial PDGFRβ expression after EPC-CM exposure. The phenotypic change was followed by increased susceptibility of the endothelium to PDGF-BB and increased angiogenesis. This new insight into the paracrine activity of EPC and the role of PDGF for the endothelium in our opinion deserves further attention and potentially offers a new pathway that can be manipulated to the end of therapeutic angiogenesis or reduction of tumor vasculature.
